# Current Perspectives in Cardiac Laterality

**DOI:** 10.3390/jcdd3040034

**Published:** 2016-12-09

**Authors:** Marina Campione, Diego Franco

**Affiliations:** 1 CNR Neuroscience Institute and Department of Biomedical Sciences, University of Padua, 35122 Padova, Italy; 2 Cardiovascular Development Group, Department of Experimental Biology, University of Jaen, 23071 Jaen, Spain; dfranco@ujaen.es

**Keywords:** left/right asymmetry, heart laterality, atrial identity, heart looping, nodal, Pitx2

## Abstract

The heart is the first organ to break symmetry in the developing embryo and onset of dextral looping is the first indication of this event. Looping is a complex process that progresses concomitantly to cardiac chamber differentiation and ultimately leads to the alignment of the cardiac regions in their final topology. Generation of cardiac asymmetry is crucial to ensuring proper form and consequent functionality of the heart, and therefore it is a highly regulated process. It has long been known that molecular left/right signals originate far before morphological asymmetry and therefore can direct it. The use of several animal models has led to the characterization of a complex regulatory network, which invariably converges on the Tgf-β signaling molecule Nodal and its downstream target, the homeobox transcription factor Pitx2. Here, we review current data on the cellular and molecular bases of cardiac looping and laterality, and discuss the contribution of Nodal and Pitx2 to these processes. A special emphasis will be given to the morphogenetic role of Pitx2 and to its modulation of transcriptional and functional properties, which have also linked laterality to atrial fibrillation.

## 1. Cardiac Asymmetry and Laterality

Cardiac development is a complex process. Mesodermal cardiac precursor cells emerging bilaterally from the primitive streak migrate through the lateral plate mesoderm in a cranial direction, delineating a primary heart field which is patterned along the anterior–posterior (A/P) and left–right (L/R) axis [1]. Soon afterwards, cardiac precursors converge towards the embryonic midline, progressively forming a linear heart tube, which in amniotes is attached to the body wall by the dorsal mesocardium (DM), thus providing the first signs of dorso–ventral (D/V) polarization. The linear heart tube initially consists of an external myocardial layer and an internal endocardial layer separated by acellular cardiac jelly. The phase of morphological left–right symmetry is very short (4–6 somites stage in mammals), as the heart tube elongates by addition of cardiomyocytes of the second heart field (SHF) at the venous and arterial poles and concomitantly starts to bend to the right, thus delineating the first sign of the looping process. Cardiac differentiation also takes place, allowing identification of a sinoatrial (SA) region, the atrioventricular canal (AVC), ventricular chambers and the outflow tract (OFT) [2,3], which will become progressively aligned into their final topological organization through a complex asymmetric remodeling process. It is important to highlight in this context that species-specific adaptations have evolved to meet specific body demands.

In mammals and birds, cardiac chamber growth and remodelling along the inner curvature is accompanied by septa formation, leading to the transformation of the linear tube into the synchronously contracting four-chambered heart, in which atrial and ventricular chamber myocardium, a conduction system and physically separate systemic and pulmonary blood streams are developed [1]. In particular, the venous pole and the atrial chambers present clear morphological differences and distinct connections to the venous system, as systemic veins drain into the right atrium (RA) and the pulmonary veins (PV) into the left atrium (LA). On the other hand, fishes present a simpler heart, composed of a single atria and a single ventricle, which are connected by a common AVC, and the OFT [2]. Amphibia and reptiles have two distinct separated atria, with a common ventricular chamber that presents different degrees of septation among distinct species [3]. Importantly, in all species, looping and asymmetric morphogenesis are necessary for correct alignment of the chambers and inlet and outlet connections, therefore for proper cardiac function.

The mature heart is thus a highly asymmetric structure. However, asymmetry within the cardiac regions is generated by different processes: the sino-atrial (SA) region is not affected by the looping process [4], thus, its morphological asymmetry, when present, must reflect original L/R differences in positional identity laid down during early development. In contrast, looping, a developmental process that occurs in all species, will drive a rotation of the AVC, the developing ventricles and the OFT, leading to a progressive shift and repositioning along the dorso–ventral axis [4]. Currently, the term “cardiac laterality” globally includes the differential L/R atrial identity and dextral looping, but the contribution of laterality to the later asymmetric remodeling processes is less appreciated.

A central issue in cardiac biology is understanding the fine mechanisms that regulate cardiac asymmetry and laterality and exploit this knowledge to unravel the molecular basis for cardiac development and disease in humans. A widely used tool to study mammalian development is the mouse model, however, in mice, the phases of early tube formation, looping and chamber differentiation occur in a very short time frame and partially overlap, therefore these events are difficult to dissect [5]. Thus, most experimental studies have been performed in chicken and zebrafish embryos, respectively, where the separated phases of early cardiac development can be clearly distinguished [2,6]. Thus, these model organisms have provided complementary information on the complex issue of laterality and asymmetry. We will describe in this review these morphogenetic events and integrate current knowledge of the role of laterality genes in their modulation.

## 2. Generation and Amplifications of L/R Asymmetries

The vertebrate body plan is characterized by highly conserved internal L/R asymmetries, globally referred as *situs solitus*, visible both in the arrangement of the heart and viscera, as well as in the anatomical organization of every organ. The establishment of a correct L/R body axis is a fundamental process in vertebrate embryogenesis, and developmental alterations in normal L/R patterning result in a wide spectrum of laterality phenotypes, which can be broadly classified as heterotaxy and *situs inversus*. *Situs inversus* is a condition in which all organs present reversal in the L/R axis. In heterotaxy, or *situs ambiguous*, asymmetries in structure and placement of organs occur stochastically due to failure to establish asymmetry or errors in relay of axial patterning. In particular, within the heart, the laterality of the SA region and looping directionality are not coupled. Heterotaxy also includes isomerism, a condition in which the L/R morphological differences in the SA region are not visible [7,8,9,10], which is associated with the occurrence of several congenital heart defects (CHDs). Intriguingly, it has become evident in the last few years that some isolated forms of CHD might be the only visible manifestation of an underlying L/R patterning defect [11,12,13].

Experiments on vertebrate laterality date back to the 19th century (reviewed in [14]), but only in 1921 was it proposed that organisms had an underlying mechanism for generating asymmetry [15]. In 1990, a landmark paper by Brown and Wolpert [16] hypothesized that body asymmetry was biased in a consistent direction by the presence of a handed asymmetric molecule or structure (represented in their model by an F-molecule), which can orient the L/R axis to the preexisting A/P and D/V axis. Then, additional molecular interactions feeding off the deduced L/R vector would eventually lead to asymmetric organ development The first breakthrough paper describing asymmetric gene expression dates back to 1995 [17] and since then we have gained exponential information on the molecular and cellular mechanisms driving laterality (for some recent reviews see: [18,19,20,21,22,23,24]).

It is now widely accepted that establishment of body L/R asymmetry is the result of three consecutive steps. The first step is the symmetry-breaking mechanism, which generates the L/R axis and orients it with the A/P and D/V axes. The second step involves amplification of the initial L/R difference and their spatial propagation though the embryo. Finally, within the third step these signals are locally interpreted and converted by the organ primordia into anatomical asymmetry.

In the most established model of symmetry breaking, mainly studied in mice, initial L/R asymmetry is generated by a coordinated leftward flow of the extraembryonic fluid, generated by the rotational movement of cilia at the L/R organizer, identifiable as the posterior part of the notochord (i.e., the node in mice; reviewed in [23,25]). The cilia are tilted towards the posterior part of the node, where an A/P and D/V axis can be recognized, due to the asymmetric positioning of their basal body. These cilia have thus an intrinsic chirality, and the direction of their rotation determines the L/R axis [26]. Interestingly, in this model, the motile cilia fulfil the criteria of the “F” handed L/R asymmetric generator predicted by Brown and Wolpert [16]. This model is supported by experimental data in zebrafish, amphibians and in some mammals (mice and rabbits) [27,28,29,30]. Importantly, mutations affecting ciliary biogenesis, motility or sensory functions in mice and humans lead to abnormal left–right development and cardiac laterality [31,32,33,34], thus supporting the hypothesis that cilia and nodal flow can be drivers of laterality.

The most immediate target of flow is the Nodal antagonist Cerl2 in mouse (or its homologues in *Xenopus* and zebrafish), whose mRNA is more rapidly degraded at the left side of the node, thereby leading to increased expression of Nodal [35,36,37]. In parallel, a localized left increase of intracellular Ca^2+^ is mediated by sensory cilia located at the periphery of the node [18,22,25,38]. These early asymmetries must be transferred to the left lateral plate mesoderm (LPM), to initiate there the propagation of the asymmetrical information. This is probably the result of direct Nodal transport via the extracellular matrix (ECM) [39] and transmission of asymmetric Ca^2+^ signals via gap junction communication within the gut endoderm [40]. Additionally, rapid diffusion of Nodal to the LPM has been documented in zebrafish, via a proprotein convertase-mediated modulation of Nodal signaling range [41].

Noticeably, L/R asymmetry is set in some animals long before cilia are present, or without the presence of motile cilia (chicks), or in complete absence of cilia (pig) [42]. Thus, the “ciliar flow” cannot be a considered a universal model for L/R asymmetry break. In particular, in chicken embryos, oriented leftwards cell migration around the node at early gastrulation generates morphological asymmetry of the node itself [42]. Such morphological asymmetry is visible before the onset of an asymmetrical cascade in a wide number of genes, including the Nodal gene [43], which is generated around the node as a passive effect of the rotational movements ([42], reviewed in [44]) These early molecular asymmetries are subsequently amplified, eventually converging into Nodal activation in the left LPM (reviewed in [44]).

An alternative model, the “ion flux” hypothesis, although recognizing the importance of ciliar flow, proposes that L/R symmetry breaking occurs much earlier. This model proposes that, in embryos at the 2–4 cell stage, the chiral structure of microtubules and actin cytoskeletal components leads to asymmetric distribution of ion pumps, thus leading to the generation of a L/R electrochemical gradient (reviewed in [20,21]). These steps have completely been elucidated in *Xenopus* embryos, but several of these components have been identified in other species, including chickens [20].

The second step in laterality determination is the amplification of the L/R differences and their propagation through the embryo. In this context, asymmetric Nodal activation in the left LPM is crucial. However, Nodal is only transiently expressed [45,46], due to the activation of its inhibitors Lefty2 in the same region and of Lefty1 in the midline, which also prevents Nodal activation in the right LPM [47,48,49]. Thus, Nodal and Lefty act respectively as diffusible activator and inhibitor molecules and together constitute a self enhancement and lateral inhibition system (SELI) which provides robustness to laterality [48]. In this context it is important to highlight that mice lacking or unable to respond to Nodal signaling in the LPM showed a randomized direction of looping (reviewed in [19]), in line with its relevance as a left-side determining molecule. It is therefore not surprising that Nodal activation is highly controlled by a number of molecules in the left and right LPM, whose identity is only partially conserved in the different species [19,44].

Eventually, Nodal signaling in the left LPM upregulates the expression of the homeobox gene Pitx2 [50], which is expressed also in the developing organs, including the heart, and has been proposed to transduce the “left” molecular information into asymmetric cardiac morphogenesis, as we will discuss in the following paragraphs. The expression of this set of genes defines the so called “Nodal signaling cascade”, which is a conserved feature of distinct species, from fish to mice [51], with the noticeable exception of chickens, where Lefty2 expression is missing [44].

## 3. Cardiac Looping Onset

The third and final step in laterality determination is the local “interpretation” of the developing organs into anatomical asymmetry. In the heart, the first sign of morphological asymmetry is visible as the onset of dextral rotation at the caudal part of the heart primordium, which prefigures the early SA region. The correct directionality of looping is fundamental to establishing normal atrio-ventricular concordance.

To date, the most detailed road map on the cellular and molecular mechanisms driving early cardiac asymmetries has been provided by the zebrafish model (reviewed in [2,52,53]). In zebrafish, the bilateral cardiac progenitors initially converge into a symmetric heart disc, in which atrial cells are positioned more externally and ventricular cardiomyocytes internally [2,52,53]. Symmetry breaking can then be divided into two clearly distinguishable steps: the first, called jogging, is characterized by leftwards and cranial displacement of atrial cardiomyocytes [2,52] and concomitant involution of ventricular myocardial cells. This process leads to an initial clockwise rotation of the heart cone and transforms it into a small tube. Jogging is driven by cell polarity genes and requires extracellular matrix (ECM) component deposition, which are both under the control of the Nodal laterality pathway [54,55]. More recent data highlight the complexity of this process since differential cell migration is regulated by integrated, but different, actions of Nodal signaling in the left myocardial region and of BMP signaling in the right endocardial region, via a common target, the transcription factor FoxH1 [56].

This initial symmetry breaking process is followed by d-looping, which repositions the atrial caudally and the ventricles more anteriorly, and delineates the position of inner and outer curvatures [54]. Normally, the direction of jogging prefigures also the directionality of heart looping, suggesting that laterality genes control both steps. Noticeably, fish heart tubes, isolated and cultivated in vitro, undergo d-looping [57] and this process could be prevented by blocking actin polymerization, or the activity of non-muscle myosin II (NMHCII), an actin binding protein which plays a fundamental role in cellular morphogenesis, adhesion and polarity by modulating the contractile cytoskeleton [58]. Similar findings have also been described in chicken hearts [59], thereby suggesting that common “cytoskeletal-based” mechanisms drive early cardiac asymmetries in different species.

Importantly, d-looping also occurs in Nodal pathway mutant fish hearts, although at a reduced rate [57]. In this context, it has been shown that the cytoskeletal gene *α-actin1b* is asymmetrically expressed in the early myocardium and that its expression is modulated by Nodal [57]. Altogether, these data indicate that nascent cardiomyocytes possess an intrinsic bias to laterality, based on their cytoskeleton composition, which is robustly reinforced by the early action of the Nodal pathway. Thus, robust early cardiac asymmetry is possibly generated by the integration of an intrinsic cardiomyocyte cellular program and a local modulation of molecular signals, initially driven by action the Nodal laterality pathway.

## 4. Cardiac Morphogenesis and Asymmetric Remodeling

It is often unappreciated that cardiac looping is overall a long process, involving not only the initial break of symmetry, but also bending and rotational movements. Indeed, looping can been divided into several steps [4]: the initial phase, characterized by ventral bending and rightwards rotation of the SA region, which is accompanied by cardiac growth at both poles. Then, looping progression is accompanied by chamber growth at the outer curvature and leftwards shifting of the AVC, followed by progressive repositioning of the ventricles towards the atria. Finally, the OFT shifts to the left with a concomitant twist of 180°. All these movements are crucial to allow correct atrial and ventricular septation and for proper alignment of the forming base of the aorta and pulmonary artery (PA) with the left ventricle (LV) and the right ventricle (RV), respectively. From this description we can appreciate that looping progression is intimately associated with asymmetric remodeling, and that the poles of the heart, the AVC and the ventricles are differentially remodeled along the L/R and D/V axis. This indicates that the driving cellular and biophysical mechanisms, as well as the underlying molecular regulators act differentially along the developing heart.

It is unavoidable that even minor defects in these morphogenetic events can result in heart defects. In particular, failure of leftwards shift of the AVC can cause double inlet left ventricle (DILV), a condition in which the blood from both atrial chambers flows into the LV. If DILV is accompanied by atrioventricular septal defects, this will result in common AVC. Moreover, impairment in the correct alignment or rotation of the OFT relative to the ventricles can result respectively in double outlet right ventricle (DORV), a condition in which the LV has no outlet and the RV communicates with the aorta and pulmonary artery (PA), or in transposition of the great arteries (TGA), a condition in which the alignment of the aorta and the PA with the corresponding ventricle is inverted.

Cardiac remodeling is accompanied by cell ingression into the heart. A subset of migrating neural crest cells invades the endocardial cushions of the outflow tract, playing a critical role in normal OFT septation (reviewed in [60]). To date, no evidence exists on the asymmetric contribution of the neural crest to the heart. At approximately the same stage, the heart recruits epicardially-derived cells that will contribute to the connective tissue of the adult ventricles and the coronary vasculature (reviewed in [61]). The epicardial lining of the heart develops from the proepicardium (PE), a cauli-flower transient structure located with the junction between the cardiac venous pole and the septum transversum [61]. In mice, the PE initially develops as two symmetrical structures that eventually fuse, leading to a single PE structure [62]. In contrast, in chickens and *Xenopus* only the right-sided epicardium develops, whereas the left part remains in a rudimentary state, which is reflected by asymmetrical L/R gene expression at the PE [62]. Intriguingly, experimental manipulation has proven that asymmetric development of the PE in chickens is controlled by a right-sided molecular pathway involving FGF8/SNAI1 [63]. These results indicate that the right side of the body can also harbor instructive signaling pathways involved in cardiac development.

Needless to say, cardiac remodeling is tightly controlled by the integrated actions of multiple transcription factors and signaling pathways. The detailed description of their mechanisms of action has been excellently reviewed [64,65,66,67,68] and is beyond the scope of this work. Here, we will rather focus on the cellular mechanisms driving asymmetric remodeling, and their potential link with the mediator of L/R signaling in the heart, i.e., the Pitx2 gene, will be discussed in the following paragraphs.

Several studies have shown that coordinate regional changes in cardiomyocyte size, shape and proliferation rate underlie cardiac remodeling. In the early looping chicken heart, cardiomyocytes at the ventral part of the ventricles increase in size and proliferation rate, thus indicating D/V differences in the morphogenetic axis [6]. Similar studies in mice have shown that the developing heart has a highly anisotropic cellular organization. In particular, retrospective clonal analysis of cardiomyocyte growth has revealed local variations in size and shape of the clones, generated by different rates of mitosis [69]. Noticeably, clonal shape is oriented in relation to heart morphology, thus suggesting that oriented cell mitosis and local differences in proliferation rate could be major drivers of cardiac remodeling and shape [69]. It is additionally recognized that cardiomyocyte-originated physical forces can modulate cardiac shape. In humans and mice, cardiac remodeling defects can be caused by mutations in several components of the contractile apparatus (reviewed in [70,71]). In zebrafish embryos, cardiomyocytes at the inner and outer curvature present differential morphology, and genetic mutations which impair the contractile apparatus lead to morphogenetic defects by impairing cardiomyocyte shape [72]. Importantly, in the developing heart, cardiomyocytes present a precise spatial organization, and crosstalk between neighboring cells is modulated by the extracellular matrix (ECM), which integrates and transmits mechanical forces to the entire heart.

The role of blood flow in shaping the heart is also widely recognized (reviewed in [73]). Hemodynamic forces lead to mechanical stress on cardiomyocytes, thereby impacting on their gene expression and cellular differentiation, as shown in mouse and chicken embryos [74,75,76]. In zebrafish, genetic mutations which affect blood flow lead to morphogenetic defects mostly due to altered cardiomyocyte shape [72]. Importantly, hemodynamics also drive the asymmetric remodeling of the branchial arch system, which underlie the asymmetric formation of the aortic arch [77].

Altogether, the presented data indicate that asymmetric remodeling during cardiac morphogenesis is mostly due fine modulation of multiple cellular properties of cardiomyocyte and of their crosstalk, with the contribution of external forces such as hemodynamics.

## 5. The Differential L/R Atrial Identity

In vertebrates with two atria (amphibians, reptiles, birds and mammals), the left and right atria are morphologically different and have distinct connections to the venous return system. In humans, the RA is characterized by the presence of extended pectinate muscles, drainage of the systemic veins and by the presence of the sinoatrial node (SAN) associated with the right superior caval vein (SCV). In contrast, pectinate muscles are less prominent in the LA, which additionally presents a smooth walled dorsal wall and is characterized by the drainage of PV. In mice, morphological differences between the LA and RA are less prominent, but differential venous drainage is conserved. In right atrial isomerism (RAI) the left auricle is identical to the right one, the atrial septum is mostly absent, the pulmonary vein (PV) and the inferior caval vein (ICV) drain into the medial part of the common atrium, the superior caval veins (SCV) enter bilaterally into the two atria, no coronary sinus (CS) is visible and two SAN can be detected [7,8,9,10]. On the other hand, in left atrial isomerism (LAI) the atrial septum is normally present, venous connections are also impaired and the SAN is missing or strongly hypoplastic [7,8,9,10]. The association between atrial isomerism (LAI and RAI, respectively) and the absence or duplication of the SAN suggests that a unique molecular pathway determines the differential L/R atrial identity by modulating both the morphology of the SA region and the asymmetric positioning of the SAN. In this context, it is important to highlight that the left–right atrial chambers and their connecting venous tributaries have also a distinct molecular identity [78] which is acquired very early in development, i.e., around E8.0–8.5 [78,79,80].

Furthermore, it is important to mention that fish hearts are characterized by a common atrium, which does not present evident L/R morphological differences, suggesting that the acquisition of a morphological L/R atrial identity is correlated with the presence of pulmonary circulation. Nevertheless, a functional right-sided SAN, identifiable by optical mapping [81] and expression of genetic markers [82] is present in zebrafish hearts, indicating the existence of a positional control by the laterality pathway.

## 6. The Role of Pitx2 in Cardiac Laterality and Asymmetric Morphogenesis

**Figure 1:**
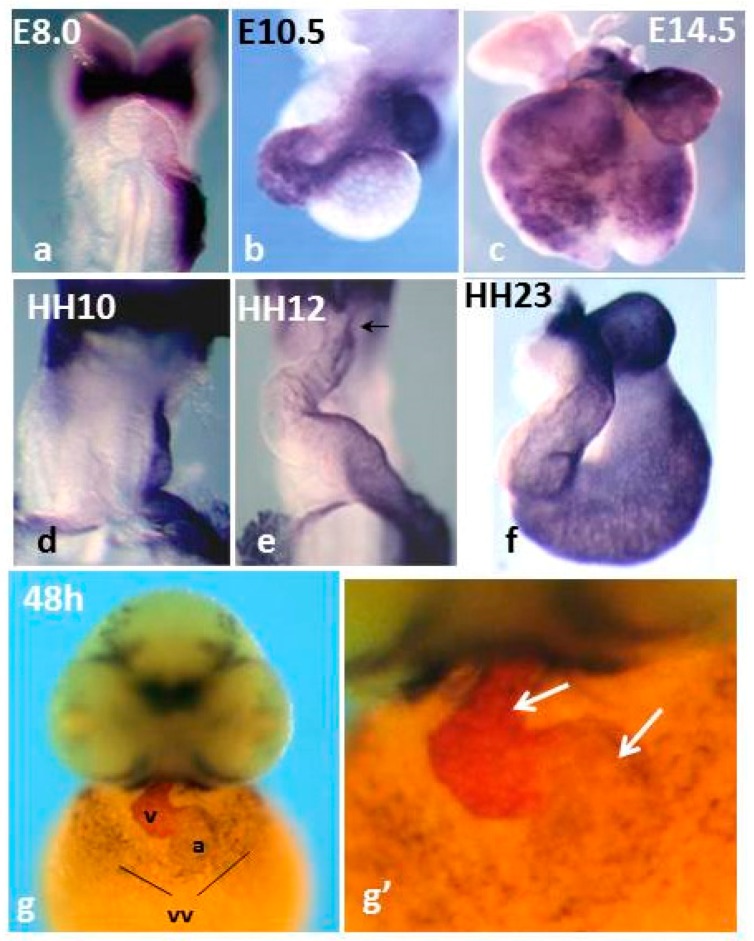
Comparison of Pitx2 mRNA expression in mouse (**a**–**c**), chicken (**d**–**f**) and zebrafish embryos (**g**,**g’**). Mice and chicken, single *in situ* hybridization (ISH); zebrafish, double ISH: Pitx2, dark blue, and mlc2v, magenta. Note that in chickens and mice, Pitx2 delineates the left heart primordium and its remodeling. In developing zebrafish hearts, Pitx2 expression delineates the common atrium and is regionalized in the ventricles. vv: vitelline veins. Panels **a**–**f** are reproduced with permission from [85].

Pitx2 is a homeodomain transcription factor of the bicoid family. From a single gene three different Pitx2 isoforms (a, b, c) are generated [83], which share a common C-portion of the protein, including the homeodomain, and differ at the N-terminal part. Pitx2 isoforms are coexpressed in several developing structures [84], but only Pitx2c is additionally expressed asymmetrically within the left LPM, the left SHF and the left linear heart tube [85,86,87]. Comparison of Pitx2c expression and fluorescent labeling-mediated fate map experiments suggested that Pitx2 can be considered as a surrogate left lineage marker in the developing heart [85], therefore its expression outlines the remodelling of the left linear heart tube. During mouse and chicken cardiogenesis, where it has been most deeply characterized, Pitx2c expression delineates the left SA region, the left AVC the ventral part of the ventricles and the left-ventral part of the OFT [85,86,87] (Figure 1), thus showing that the initial L/R asymmetry is maintained as such in the developing SA region and is converted into D/V differences in the ventricular chambers [85]. Interestingly, in zebrafish hearts, Pitx2 expression delineates the common atrium and a subset of ventricular cardiomyocytes (Figure 1). Pitx2 expression in the fish common atrium suggests that compartmentalization within the left SA region has evolved during evolution and is correlated with a role of the gene in LA identity and double circulation. In mice, Pitx2c expression is strongly down-regulated at late foetal stages, but it is still detected in adult hearts [88,89,90,91,92]. Curiously, expression of all Pitx2 isoforms has been detected in the adult mouse and human RA [89], though at low levels, in line with recent developmental data during late cardiogenesis [92].

The role of Pitx2 in cardiac laterality and asymmetric morphogenesis is clearly outlined by the complex cardiac phenotype of constitutive and myocardial specific Pitx2 knockout (ko) embryos, which display RAI, impaired atrioventricular remodelling, atrial and ventricular septal defects, DORV and TGA [93,94,95,96,97,98]. Constitutive Pitx2 ko mice additionally recapitulate distinct aspects of Axenfeld-Rieger syndrome (ARS), such as congenital eye and tooth malformations [93,96,97,98], in line with reports demonstrating PITX2 loss-of-function mutations in ARS patients [84,99]. Importantly, congenital heart defects have also been reported in ARS patients, although with low frequency [99,100,101,102,103], in line with the finding that low doses of Pitx2 are sufficient for normal cardiac morphogenesis [94]. These data support the notion of a modular and dose-dependent role for Pitx2 during different aspect of L/R asymmetry and organogenesis.

Noticeably, none of the mouse Pitx2 ko models, nor a recently published zebrafish Pitx2 loss-of-functions mutant [104] present randomization or inversion of looping, thereby supporting the notion that looping is an intrinsic property of the early heart. However, conflicting results on the role of Pitx2 in looping directionality come from gain and loss of function studies in other animal models. In fact, both Pitx2 loss of function by antisense oligo treatment in chickens [105], as well as overexpression of Pitx2 in chicken and *Xenopus* embryos [105,106,107] resulted in looping randomization. Whereas it is formally possible that the gain of function phenotype might be due to artefactual effects of ectopic Pitx2 expression, the loss of function results are more difficult to explain. It is however possible that looping directionality could be subjected to more robust control in mice and zebrafish than in chickens (and *Xenopus*), and/or thus involve additional, yet unidentified, molecular players. The implication of this hypothesis is that additional pathways acting in parallel to Nodal–Pitx2 could altogether drive looping directionality. Evidence for such genes indeed exist in mice, as it has been reported that Ablim 1 (actin binding lim protein 1) is expressed asymmetrically in the node and left LPM and is independent from Nodal and Pitx2 signaling [108]. The functional role of the Ablim1 gene needs to be proven in vivo, nevertheless its expression profile provides strong evidence for the existence of parallel pathways in the left LPM, at least in mice, which might converge on cardiac looping directionality.

Combined *in vivo* and *in vitro* findings support the notion that Pitx2 modulates molecular players and cellular events, described in the previous paragraphs, involved in looping and asymmetric remodeling. In mouse ventricles, Pitx2 expressing cardiomyocytes, located in the ventral part of the ventricles, are more differentiated with respect to myofiber organization and cellular elongation, than Pitx2-negative dorsal cardiomyocytes [87]. These differences are lost in Pitx2 ko cardiomyocytes, which present a disorganized myofiber organization and lack oriented directionality [87], thus indicating that Pitx2 modulates ventricular cardiomyocyte maturation and cytoarchitecture. In line with this, the ectopic expression of Pitx2 *in vitro* results in changes in cell morphology, and cytoskeletal actin–myosin reorganization, via the activation of the Rho GTPases Rac1 and RhoA [109]. Additionally, Pitx2 regulates the asymmetric expression of nMHCIIB in chicken hearts [106], although this has not been tested in mice.

The action of Pitx2 at the arterial pole of the heart has been deeply investigated. A Pitx2–Wnt11 pathway regulates OFT elongation by affecting ECM composition, cytoskeletal rearrangements, polarized cell movements [110] and regional proliferation [111,112]. Additionally, Pitx2 drives the counterclockwise OFT rotation [113], which leads to asymmetric blood supply to the sixth branchial arch artery (BAA) and uneven distribution of hemodynamic forces [77]. This contributes to differentially signaling of PDGFR and VEGFR2, which drive the stabilization of the left sixth BAA and regression of its right counterpart, resulting in left-sided formation of the aortic arch [78]. Thus, Pitx2 can affect cardiovascular morphogenesis by acting also on hemodynamics, although indirectly.

It is worth mentioning too that the patterns of cardiomyocyte clonal growth, which have been shown to underlie cardiac shape, appear differentially modulated in the dorsal and ventral part of the ventricles and in the left and right atrial chambers [69]. These differences have been correlated with the expression of Pitx2 [69], thus leading to speculation that initial L/R differences can modulate cardiac morphogenesis also by affecting the pattern of clonal growth. In line, also the D/V differences in cardiomyocyte size and rate in embryonic chicken ventricles [6] could outline this fact. Altogether, these data support a fundamental role of Pitx2 in modulating multiple cellular properties which drive looping and asymmetric cardiovascular morphogenesis.

The observation that Pitx2 ko mice display RAI indicates that Pitx2 directs LA morphological identity. This role seems to involve regulation of directional migration and proliferation, which occur in a critical time window around E8–8.5 [79,95]. Pitx2 deficient mouse hearts additionally present a duplicated SAN, indicating that Pitx2 confers the LA identity by regulating both the morphology of this region as well as the positional identity of this central conduction system component. In line, it has been shown that Pitx2 normally prevents the expansion of early differentiated left SAN cardiomyocytes, thereby confining the pacemaker activity to the right side [95]. Quite intriguingly, to date no gene has been able to drive the right atrial identity. Thus, right identity seems to be a “default” state of the SA cardiomyocytes, which can be turned into left only by the local action of Pitx2.

## 7. Modulation of Pitx2c Expression in the Heart: Implications for CHD

It has been widely established that Pitx2c expression is regulated by a basal P1 promoter and by an intronic enhancer (ASE), which controls the asymmetric expression of the gene. [50,114]. Pitx2 ASE contains multiple Foxh1-binding sites and an Nkx2-5 binding site, which are essential and sufficient for asymmetric enhancer activity and are evolutionarily conserved among vertebrates [114]. Foxh1–FAST binding sites function as Nodal-responsive elements and are sufficient for the initiation but not for the maintenance of asymmetric expression the heart, which is mediated by the Nkx2-5 binding site [114].

However, additional findings indicate a higher complexity in regulation of Pitx2c expression. In the *iv/iv* mouse model of heterotaxy, expression of Pitx2c in the LPM is absent, bilateral or restricted to the left [107] and correlates with the distribution of Nodal [45,107]. However, analysis of the pattern of Pitx2c expression in the *iv/iv* hearts revealed no correlation with Pitx2c LPM expression; moreover, in the same model, alterations in atrial and ventricular Pitx2c expression did not always occur concomitantly [85]. These findings indicate (1) that cardiac Pitx2 expression can be regulated, at least partially, by Nodal unrelated pathways; and (2) the existence of a modular regulation of Pitx2 gene expression in different regions of the heart. These two conclusions have been validated by independent studies. Pitx2 expression in the left SHF and the heart can be modulated by Tbx1, which can bind directly to a T-site within the ASE, as well as interact with Nkx2.5 [86]. In line, Pitx2 expression is downregulated in Tbx1 ko hearts [86]. Intriguingly, mouse embryos lacking the miRNA processing enzyme Dicer [115] display Pitx2 upregulation selectively in the OFT, thus additionally indicating a novel miRNA-mediated transcriptional modulation of the gene.

More complex results emerge from the analysis of mice lacking the transcriptional coactivator Cited2. Cited2 null mice generated in the C57/Bl6 genetic background present a partially penetrant but strong cardiac laterality phenotype, which includes RAI and looping defects, due to downregulation of the Nodal–Pitx2c pathway [116]. In line, Cited2 is required to potentiate the initiation of the left LPM expression of Nodal, by acting on the ASE element of its promoter [11]. However, Cited2, has also been shown to bind together with TFAP2 to Pitx2 P1 promoter, thus suggesting a Nodal-independent modulation on Pitx2 transcription [116]. Noticeably, in a mixed genetic background, the Cited2 null cardiac phenotype is much milder, RAI and laterality defects are not detected and normal Pitx2 expression is retained [116]. A high fat diet has been shown to increase the penetrance of cardiac laterality defects and to reduce Pitx2c expression in Cited2 null embryos, but not in wild type littermates [117]. These findings indicate that genetic modifiers and dietary factors can modulate the laterality defects and the expression of Pitx2 in Cited2 null mice.

Taken together, the implications of these results above described are twofold: (1) given the role of Pitx2 in setting the left atrial identity and in asymmetric remodeling, the possibility of a modular activation of Pitx2c could underlie the wide heterogeneity of cardiac phenotypes observed in laterality syndrome patients; (2) genetic and environmental modifiers are likely to play a role in modulating (either suppressing or enhancing) the effects of mutation in the L/R pathways, as observed in humans and in animal models [11,12,13].

## 8. Pitx2, the Molecular Identity of the SA Region and Atrial Fibrillation

Additional evidence indicates that Pitx2 does not only play a morphogenetic role in the embryonic hart, but it can additionally modulate there the molecular identity of the left SA region [87,88,95]. Thereby Pitx2 prevents the activation of a “nodal-type” transcriptional activity, which is normally confined to the right-sided SAN [88,95], and, in line, absent or reduced expression of the gene favours abnormal conduction in this region [95]. This finding is interesting in the light of a novel set of studies that have identified Pitx2 as a candidate gene for atrial fibrillation (AF) in humans. In particular, the presence of single nucleotide polymorphisms (SNPs) on chromosome 4q25, located in a region 165 kb distal to the Pitx2 gene, has been associated with increased risk for AF [118,119]. In humans, the correlation between AF and Pitx2 gene dosage has not been definitively clarified, due to lack of uniformity in numbers and age of patients enrolled in different studies, tissue sample analysed (LA or RA) and Pitx2 isoforms detected [90,120,121]. However, adult Pitx2 heterozygous mouse hearts have increased susceptibility to AF [88,89], thus suggesting a correlation between reduced Pitx2 gene dosage and AF, at least in mice. Constitutive adult Pitx2c heterozygous and conditional Pitx2 ko mice display shorter action potential duration and lower resting membrane potential [89,90], as a result of modulation of multiple targets, including transcription factors, ion channels, calcium handling genes, microRNAs as well as other AF susceptibility genes [88,89,90,122,123,124]. Altogether, the available data suggest that Pitx2 action in regulating SA functionality is initiated during embryonic life and is then reinforced during adulthood. Importantly, recent studies have uncovered an antagonistic role between Pitx2 and Tbx5 in establishing a correct gene network to modulate adult atrial rhythm [125], thus adding a new level of complexity to the regulation of SA functionality.

## 9. Conclusions and Perspectives

The establishment and regulation of cardiac laterality is a complex issue. It is now recognized that three concatenated steps lead to cardiac laterality. Our understanding of the first step of L/R symmetry break has progressively evolved in recent years providing evidence for two models: the cilia-nodal flow and the ion flux models. Common to both of them is the concept of an intrinsic chiral asymmetry of the cytoskeleton which, through different means, initiates an asymmetrical gene cascade. Nonetheless, some exceptions exist, as in chick a morphological asymmetry, due to oriented cell migration, is detected at the node before the molecular asymmetry. Interestingly, it is known that some migrating cells possess intrinsic cytoskeletal polarity [126,127]. If this feature were operative also in chicken nodal cells, it could represent a unified L/R symmetry break system. Noticeably, emerging evidence suggests that asymmetries in the cytoskeleton are relevant for L/R development both in vertebrates and invertebrates [8,9,10].

**Figure 2:**
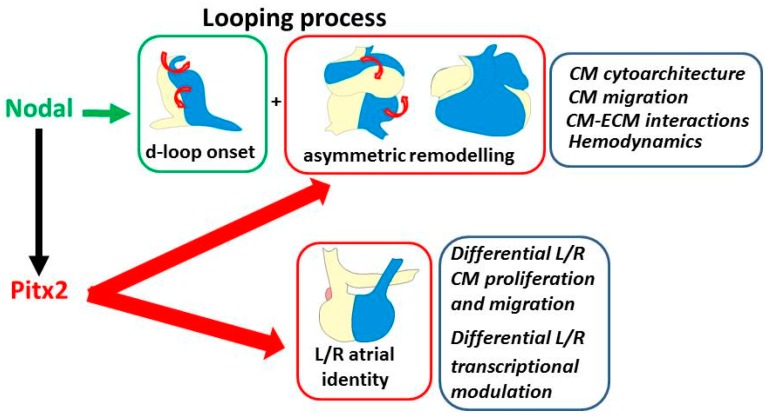
The recognized role of Pitx2 and Nodal in modulation of the two components of cardiac laterality. Top boxes: looping and asymmetric remodeling; the illustrations show the developmental repositioning of the left/right portions of the linear heart (blue/yellow color) with looping progression and, aside, the underlying driving processes. They are initially driven by Nodal in early cardiomyocytes, then reinforced by Pitx2 during development. Bottom box: left–right (L/R) atrial identity; the underlying characteristics, which are under the control of Pitx2, are indicated aside. CM: cardiomyocyte; ECM: extracellular matrix.

Cytoskeletal asymmetry is a recurrent finding in cardiac laterality. Early cardiomyocytes possess an intrinsic cellular asymmetry and current evidences in zebrafish indicate that directionality of looping is generated by the integration of an intrinsic cardiomyocyte cellular asymmetry and a local modulation of molecular signals provided by Nodal (Figure 2). Two important questions that remains to be elucidated are: (a) how cardiomyocyte asymmetry is originated; and (b) how (or whether) these cellular properties are integrated with the asymmetrical molecular cascade converging on Nodal activation. Cardiac precursor mesodermal cells emerge from the primitive streak and migrate to the lateral plate mesoderm, eventually contributing to the developing heart, and during this process they progressively receive positional cues. It is well-documented that several cell lines, including muscle cells, present an intrinsic left-right chirality/asymmetry, based on cytoskeletal dynamics, which can be amplified by external mechanical signals [127,128]. A similar mechanism might also be applicable to the developing cardiomyocytes. In this line of thinking, evidence in zebrafish cardiogenesis suggests that the intrinsic asymmetric bias in early cardiomyocytes is progressively amplified by exposure to a complex external environment, and both these aspects are modulated by laterality genes. It would be important to explore if similar mechanisms also account for cardiomyocyte asymmetry in chicken and mice.

Laterality action extends beyond the initial phase (Figure 2). We have seen that looping is overall a long and complex progress, required to align the differentiating cardiac chambers into their definitive topology. Differences in cardiomyocyte shape, size and proliferation, as well as hemodynamics play a crucial role during asymmetric morphogenetic remodeling, and Pitx2 has been shown to modulate all these steps. However, the detailed mechanisms of action of Pitx2 have only been partially elucidated. It has been shown that Pitx2 is a target of Tbx1 and that the two genes genetically interact in the heart [86]. A major missing point is the elucidation of the hierarchical relationships and genetic interactions between Pitx2 and the large number of additional transcription factors involved in early cardiogenesis.

Cardiac laterality does not involve only looping progression, but it also implies the onset of differential L/R atrial identity, which is under the control of Pitx2. Importantly, hallmarks of laterality extend beyond morphogenesis, but additionally involve the molecular modulation of gene regulation in the developing and adult SA region, thereby providing a link between Pitx2 and modulation of cardiac functionality. Pitx2 can regulate the positional identity of the SAN, however we have to consider that the entire cardiac conduction system presents an asymmetric distribution. Thus, it remains to be established if laterality accounts also for the asymmetric formation of other components of the cardiac conduction system such as the AV node, the left and right AV bundle branches and the His-Purkinje system [129]. Morphological analyses of the conduction system in human isometric hearts [10] and in the L/R mutant mouse model *iv/iv* [130] suggest that L/R cues can influence AV node formation.

Our current knowledge on cardiac laterality, based on studies in animal models, should be helpful to unravel the molecular basis for cardiac development and disease in humans. Current evidence demonstrates that defects in onset or relay in positional information during development cause cardiac laterality defects such as isomerism and/or *situs inversus*. Importantly, cardiac phenotype caused by mutations in genes within the L/R cascade can be modulated by both genetic and dietary/environmental factors. Thus, it is important to highlight that some isolated CHDs can indeed be the only manifestations of a laterality disease. Furthermore, the finding that Pitx2c expression can be modulated independently of Nodal open novel pathways and links between Pitx2, laterality and distinct CHDs.
